# Patterns of recruitment and injury in a heterogeneous airway network model

**DOI:** 10.1098/rsif.2015.0523

**Published:** 2015-10-06

**Authors:** Peter S. Stewart, Oliver E. Jensen

**Affiliations:** 1 School of Mathematics and Statistics, University of Glasgow, Glasgow, G12 8QW, UK; 2 School of Mathematics, University of Manchester, Manchester, M13 9PL, UK

**Keywords:** airway recruitment, mechanical ventilation, respiratory distress syndrome

## Abstract

In respiratory distress, lung airways become flooded with liquid and may collapse due to surface-tension forces acting on air–liquid interfaces, inhibiting gas exchange. This paper proposes a mathematical multiscale model for the mechanical ventilation of a network of occluded airways, where air is forced into the network at a fixed tidal volume, allowing investigation of optimal recruitment strategies. The temporal response is derived from mechanistic models of individual airway reopening, incorporating feedback on the airway pressure due to recruitment. The model accounts for stochastic variability in airway diameter and stiffness across and between generations. For weak heterogeneity, the network is completely ventilated via one or more avalanches of recruitment (with airways recruited in quick succession), each characterized by a transient decrease in the airway pressure; avalanches become more erratic for airways that are initially more flooded. However, the time taken for complete ventilation of the network increases significantly as the network becomes more heterogeneous, leading to increased stresses on airway walls. The model predicts that the most peripheral airways are most at risk of ventilation-induced damage. A positive-end-expiratory pressure reduces the total recruitment time but at the cost of larger stresses exerted on airway walls.

## 1. Introduction

An accumulation of liquid in the periphery of the lungs can induce airway occlusion and collapse, inhibiting gas exchange [1]. Pulmonary surfactant reduces the surface tension of the air–liquid interface, enabling the inspiration of gas to overcome adhesive forces and recruit the airway network, facilitating normal respiration. However, in surfactant-deficient neonates, the surface tension of the air–liquid interface remains high, so these resistive forces dominate and the passage of air remains obstructed, leading to acute respiratory distress syndrome (ARDS), where the resulting lack of oxygen can lead to severe and potentially fatal consequences if not treated efficiently; ARDS accounted for 2.1% of infant deaths in the USA in 2010 [2]. ARDS can also occur in adults as a result of many disorders or injuries [3] where the mortality rate is greater than 40% [4].

Treatment of respiratory distress in neonates proceeds by a combination of surfactant replacement therapy (SRT) [5,6], where exogenous surfactant is injected into the lungs to reduce the surface tension, and mechanical ventilation, where air is forced into the lung to recruit airways and promote gas exchange [7,8]. However, this process can exert significant stresses on the airway wall, which can in turn lead to ventilator-induced lung injury (VILI) [9]. Typical ventilation protocols involve either a prescribed airway pressure, with a peak pressure of 30–40 cm H_2_O, or a prescribed tidal volume, typically 10–15 ml kg^−1^ of a healthy individual of equivalent age, although lower tidal volumes (6–10 ml kg^−1^) can reduce lung stretch but result in cyclic recruitment/derecruitment of the peripheral airways [10].

Theoretical studies of airway recruitment fall broadly into two categories. On one hand, it is instructive to consider the local processes involved in recruiting a single airway to obtain a detailed understanding of the stresses exerted on the epithelial cells lining the airway walls. Alternatively, one can take a global approach to study the recruitment of airway networks to characterize lung compliance and examine inter-dependency between distant parts of the network.

Single airway studies consider a gas bubble advancing into a collapsed liquid-filled flexible channel or tube. They include bench-top experiments [11,12] and detailed computational models [13–15]. Across these approaches, a unified picture emerges: steady recruitment can only occur once the airway pressure exceeds a threshold, beyond which the walls peel apart rapidly; the airway wall can be tapered close to the bubble tip, with only a small amount of fluid trapped ahead of the meniscus. In some circumstances, the airway remains uncollapsed but completely flooded with liquid, in which case recruitment is well modelled by the propagation of a bubble in a uniform liquid-filled tube [16]. Bench-top experimental models of this system elucidate the stresses exerted on the epithelial cells lining the airway walls during recruitment [17,18]; cell damage appears to correlate to gradients of normal stress across the bubble tip [19,20], but an excess of pulmonary surfactant can be shown to eliminate the damage [21]. Peeling apart the airway walls during recruitment can also generate substantial mechanical stresses on the alveoli, a possible route to VILI [22].

Simple network models elucidate the global mechanical properties of the lung from the pressure–volume characteristics during ventilation [23,24]. In these models, lung units (such as airways and alveoli) are typically assigned a threshold opening pressure, with recruitment occurring once the airway pressure exceeds this threshold. Suki and co-workers [25–27] predicted avalanches of recruitment when threshold opening pressures for lung units are sampled at random, with a large number of units opening in quick succession once the critical opening pressure has been exceeded in the most proximal airway. Other network models (which exhibit similar cascade dynamics) address the propagation of a train of liquid plugs through a branching microfluidic network [28]. Models for propagation of liquid plugs through airway networks also elucidate the rate of surfactant spreading in SRT [29].

This paper considers, for the first time, a composite between these two approaches, constructing a theoretical model for mechanical ventilation of an initially flooded network of airways incorporating a detailed understanding of the mechanics of single airway recruitment [14] and the influence of airway wall elasticity and recruitment across bifurcations (challenges identified by Amin & Suki [30]). To capture lung inhomogeneity [31], both within and between individuals, the network is assumed to have a prescribed statistical distribution of geometric properties, sampling the cross-sectional area of each constituent airway from a normal distribution about a given mean for that generation. In particular, the model considers a network representing generations 11–16 of the adult human lung and incorporates a terminal acinus at the end of each branch; this small network also provides a simple representation of a neonatal lung. Sophisticated computational models have been constructed to understand alveolar dynamics in ARDS [32], but here we model the acinar mechanics using a simple relationship between the airway pressure and acinar volume based on the Salazar—Knowles relationship [33]. A large number of repeated simulations are used to estimate the variability in possible outcomes, as well as to identify features arising specifically because of intra-subject inhomogeneity.

The use of random (Gaussian) distributions of airway properties models three forms of uncertainty in the problem: first, the uncertainty in the physical properties of an individual's airway network (leading to a distribution of possible outcomes); second, the intra-individual variability, revealing physical features emerging from spatial variation across the lung structure (such as avalanche behaviour [25–27]); third, the distribution of outcomes over a population of individuals. In the absence of detailed measurements of the variability of airway properties within and across individuals, our focus here is on understanding intra-individual variability. The mathematical model presented in §2 is strongly nonlinear, leading to significant interaction between different sources of uncertainty in the model (e.g. airway properties and initial degree of collapse), making some of the predictions reported in §3 strongly non-Gaussian. The model is used to investigate optimal recruitment strategies for mechanical ventilation using a prescribed tidal volume, seeking to maximize recruitment while minimizing damage to the lung tissue through excessive mechanical stress.

## 2. Material and methods

**Figure 1.**
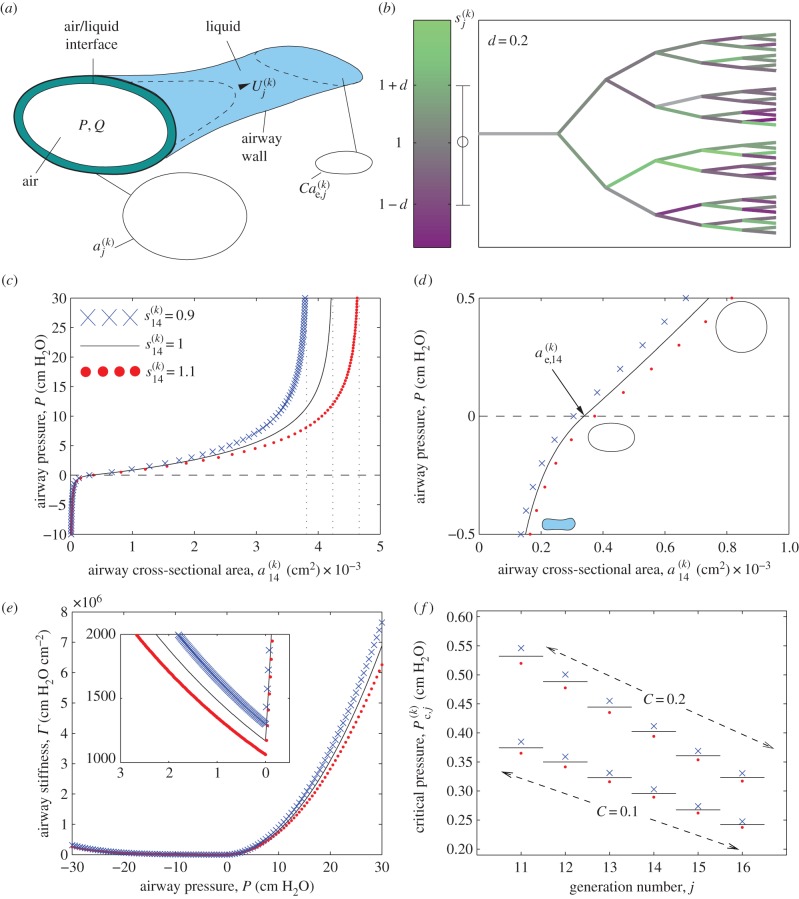
Set-up of the mathematical model: (*a*) sketch of an individual airway during recruitment; (*b*) typical airway tree where individual airways are shaded according to their value of 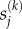, where the colour bar highlights the distribution of shading and the error bar shows 1 s.d. from the mean (darker shades represent narrower, stiffer airways); (*c*) ‘tube law’ for generation 14 showing the transmural pressure as a function of airway cross-sectional area, where 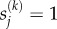 (solid line). Also shown are the cases where 
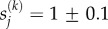
; (*d*) close-up of the ‘tube law’ for generation 14; (*e*) airway stiffness in generation 14 for the three cases shown in (*c*), with the inset showing a magnified view; (*f*) threshold opening pressure for airways in each generation of a homogeneous airway network for 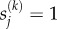 (solid line), 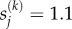 (filled circles) and 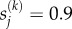 (crosses) for two levels of initial airway collapse (*C* = 0.1 and *C* = 0.2).

Airway recruitment is the process by which a finger of air advances along an airway that is flooded with liquid and possibly collapsed, held occluded by the interfacial surface tension *γ* and viscosity *μ* of the liquid. Air is forced into one end of the airway at either fixed pressure *P* or fixed flow rate *Q*, thereby advancing into the airway at speed *U*, as sketched in figure 1*a*. When *Q* is prescribed, the airway pressure is determined from the continuous increase in air finger volume and the compliance of the tube.

Previous theoretical models [13,14] predict individual airway recruitment by a rapid ‘peeling’ motion (where *U* increases with *P*, as seen experimentally [11]), provided the airway pressure *P* exceeds a threshold *P*_c_ dependent on material parameters. While sophisticated computational models have been used to predict this behaviour [13,15], simpler compartmental (or lumped-parameter) models based on coupled ordinary differential equations can be sufficient to describe the recruitment process. Such an approach is adopted here (see the electronic supplementary material), building on a model [14] which was validated (in its simplest form) against full computational simulations. It is assumed that recruitment of a particular airway halts when the airway pressure falls below the threshold opening pressure for that airway, and only restarts once the pressure has risen above this threshold again.

A segment of the periphery of the lung is modelled as an *N*-generation airway network, bifurcating dichotomously but with heterogeneous airway properties, which is assumed to be initially collapsed and flooded with liquid (a condition characteristic of an infant's first breath, or a severely diseased lung). A typical network is shown in figure 1*b* for which *N* = 6, running from generation *j* = 11 to generation *j* = 16 of an adult lung. The periphery of an adult lung is also used here as a simple analogue of a neonatal lung.

In this model, the airway pressure *P* is assumed to be controlled by volume changes in the ventilator and the open airways, so the influence of the surrounding tissue pressure (controlled by muscular action and necessary for normal respiration) is ignored. The stiffness of the airway wall is estimated using the model of Lambert *et al*. [34], which describes how changes in transmural (internal–external) pressure influence the cross-sectional area 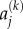 of airway *k* in generation *j* via an elastic ‘tube law’, constructed using anatomical data for generations 0–16 of the human lung [35] and expressed as function of a parameter 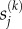 (≥0) for that airway, used to represent geometric and material properties (see the electronic supplementary material for details). To account for natural variability in individual airways across a generation, the parameter 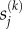 is sampled from a normal distribution with unit mean and standard deviation *d*. Airway properties (parametrized by 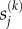) are sampled from independent distributions in this study, representing patterns of disorder with no long-range spatial correlation, although our model does assume strong correlation between geometric and mechanical properties of individual airways, as embodied in the Lambert *et al.*’s tube law [34]. To illustrate, the tube law for generation 14 is plotted as the solid line in figure 1*c* (with close-up around *P* = 0 in figure 1*d*) showing the pressure area relation *P* = *F*(*a*) for generation-14 airways with 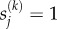. The slope of this graph, *Γ* = d*F*/d*a*, is proportional to the airway stiffness, as plotted in figure 1*e*, and inversely proportional to the airway compliance; a small increase (decrease) in 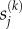 leads to a reduction (increase) in the slope and therefore a decrease (increase) in airway stiffness, as illustrated with the red filled circles (blue crosses) in figure 1*d*,*e*. Thus, heterogeneity in airway properties is regulated by variation in a single parameter 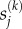 that alters the degree of maximal extension (figure 1*c*), the equilibrium area (figure 1*d*) and the stiffness (figure 1*e*).

This ‘tube law’ model is modified to accommodate an additional jump in pressure across the airway wall due to longitudinal tension *T*. Following [36], the model incorporates a simple relationship between the airway pressure and the recruitment velocity of each airway in the network, provided the airway pressure exceeds a critical (yield) pressure 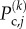 for airway *k* in generation *j*, which depends on local material properties and which incorporates dependence on the surface tension and viscosity of the airway liquid. This critical pressure is illustrated in figure 1*f* for each generation of a homogeneous airway network, where the critical pressure falls with each subsequent generation. Increasing the parameter 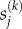 for an individual airway (representing a wider, more compliant airway) leads to a decrease in the corresponding yield pressure (figure 1*f*).

Model parameters are chosen based on measurements [37] and estimates given in previous studies [21] (table 1). Before recruitment is initiated, each airway in the network is uniformly collapsed to 100*C*% of its equilibrium cross-sectional area 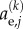 (obtained from [35] and marked in figure 1*d* for generation 14), where the parameter *C* is held constant across the network. These assumptions result in a homogeneous network compliance before ventilation is applied; a typical network is shown in figure 1*b*, constructed using anatomical data [35] but taking no account of geometrical features such as branching angles. The shading represents the value of 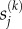 across the network, with paler shades representing more compliant airways. This distribution of airway collapse induces a variability in the initial liquid pressure through the flooded network which drives flow on long timescales, but this slow readjustment is ignored here. The yield pressure is lower for airways that are more strongly initially collapsed (figure 1*f*), reflecting greater stored elastic energy that can be released as an airway is recruited.

**Table 1.** Parameter choices for the mathematical model.

parameter	value	units	source
*T*	3000	dyn cm^−1^	[21]
*γ*	30	dyn cm^−1^	[38]
*μ*	0.01	dyn s cm^−2^	[21]
*K*_A_	0.14	cm H_2_O^−1^	[24]
*V*_A_	0.0778	cm^3^	[39]
*P*_A_	2	cm H_2_O	[40]

In the simulations reported below, attention is focused on the recruitment of a six-generation network of collapsed flexible airways using parameter values for generations 11–16 of an adult human lung. The proximal airways are assumed open throughout. Downstream of each airway in generation 16 is a flexible acinus of maximal volume *V*_A_ and compliance *K*_A_ mimicking the downstream respiratory bronchioles and the alveoli. Each acinus is assigned an acinar opening pressure (AOP), denoted as *P*_A_, and pressure–volume changes in this compartment are modelled using a modified Salazar—Knowles relationship [33].

As each airway is recruited, local liquid flow close to the tip of the advancing air finger exerts a stress on the epithelial cells lining the tissue; Gaver and co-workers demonstrated that the most likely cause of cellular damage is the large gradient of liquid pressure across the bubble tip [17,19,20]. Accordingly, the model is used to estimate the maximal pressure gradient, denoted 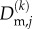 for airway *k* in generation *j*, across each tip of the advancing air finger as an estimate of damage to the airway wall. Two pertinent quantities are examined in the model simulations below: the maximal pressure gradient exerted across the entire network during a recruitment manoeuvre, denoted 
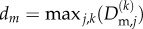
; and the maximal pressure gradient in an individual airway relative to the mean maximal pressure gradient across that generation, denoted 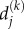.

For a given tidal volume, there is a corresponding airway pressure for which the model predicts recruitment of generation 11 at constant speed. For example, *V*_T_ = 10 ml kg^−1^ for a network collapsed to 20% of its equilibrium cross-sectional area (*C* = 0.2) corresponds to pressure *P* = 0.822 cm H_2_O. In the absence of an applied positive-end-expiratory pressure (PEEP), this constant-speed recruitment pressure is used as the initial airway pressure for the simulations discussed below. When using a PEEP (§3.1), this pressure is instead used as the initial airway pressure.

The values chosen for the parameters are listed in table 1. Additional detail on the model is provided in the electronic supplementary material.

## 3. Results

**Figure 2.**
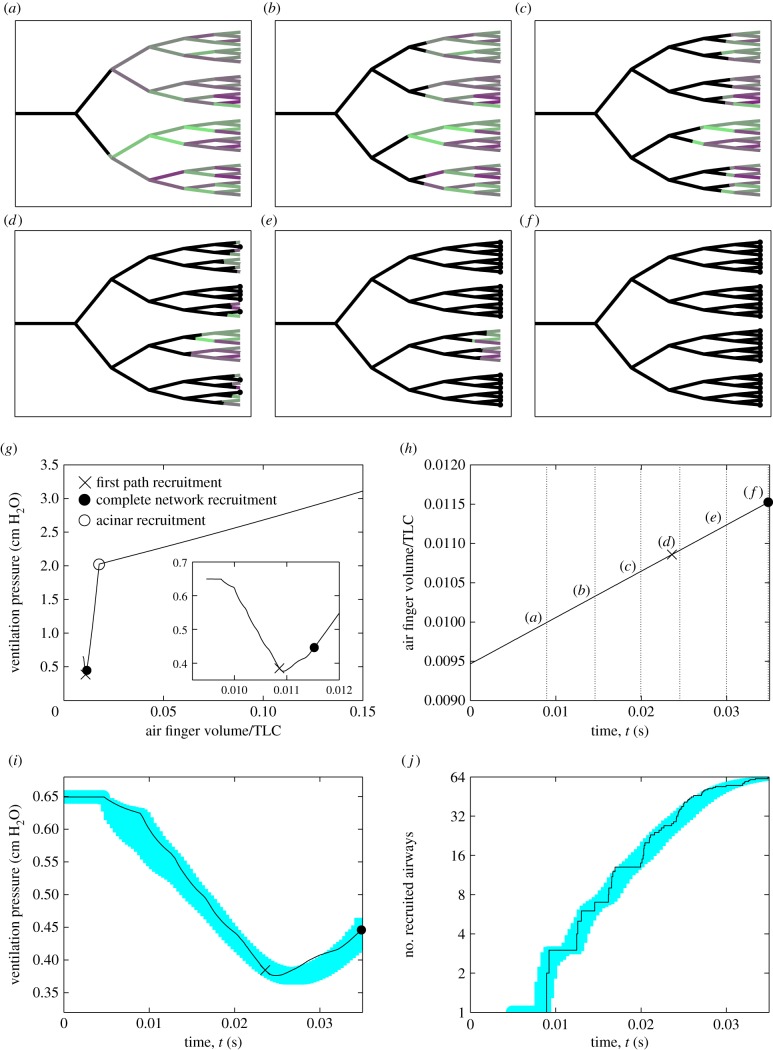
Typical recruitment event with fixed tidal volume *V*_T_ = 10 ml kg^−1^ for *C* = 0.1: (*a–f*) snapshots of recruitment of the network shown in figure 1*b* at six approximately equally spaced time intervals (labelled on (*h*)), where black lines illustrate the air finger; (*g*) pressure–volume curve during ventilation, with a close-up during recruitment (inset); (*h*) time-trace of air finger volume during recruitment; (*i*) a time-trace of airway pressure during recruitment; (*j*) time-trace of the number of recruited airways. The shaded regions in (*h–j*) correspond to 1 s.d. from the mean for 100 repeated simulations. In (*h–j*), the cross represents the time the first passage through the network is recruited, the filled circle marks complete recruitment and the open circle shows the first opening of the acini. Dotted lines on (*g–i*) represent the times for the six snapshots shown in (*a–f*). Other parameters for this simulation are chosen as in table 1.

To assess the dynamics of an individual recruitment event, figure 2 illustrates ventilation of the collapsed airway network illustrated in figure 1*b*. The electronic supplementary material, movie, provides an animation of the process. Figure 2*a–f* provides six snapshots of air finger propagation through the network (solid black line) at approximately equally spaced time intervals, while figure 2*g–j* illustrates various signatures of the recruitment event, some of which might be detected clinically, including the pressure–volume trace (figure 2*g*), a time-trace of the total lung volume (figure 2*h*), a time-trace of the airway pressure (figure 2*i*) and a time-trace of the current number of recruited airways (figure 2*j*). Because the air finger volume increases linearly in time in all cases (figure 2*h*), the corresponding pressure–volume curve (figure 2*g*) has an identical shape to the time-trace of airway pressure (figure 2*i*). In these figures, the calculated lung volume (figure 2*h*,*i*) extrapolates the small network considered here to reflect generations 11–16 across the whole lung and also includes the proximal airways and the acini. In this example, the parameter 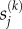 for each airway is sampled from a normal distribution with modest variability (*d* = 0.2), which translates into a distribution of airway stiffness, and each airway is initially uniformly collapsed to 10% of its equilibrium cross-sectional area (*C* = 0.1). The network is ventilated with a prescribed tidal volume of *V*_T_ = 10 ml kg^−1^ applied over a time interval *T*_Q_ = 2.5 s, although recruitment occurs within 0.04 s in this example. Heterogeneity in the airway properties ensures that the spatial distribution of the air finger quickly becomes asymmetric (figure 2*c–f*), with stiffer airways (darker shading) opening more quickly as they have more stored elastic energy (decreasing 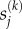 corresponds to an increase in the recruitment speed despite a corresponding increase in the yield pressure, figure 1*f*). In this example, the air finger is spread across at most three generations of airways at any instant in time, so the asymmetry remains localized. As an airway is recruited, the rate of elongation of the air finger is rapid due to the release of elastic energy from the collapsed airway walls. The cross-sectional area of the recruited airways must reduce to ensure the gain in finger volume is matched by the prescribed air flow, leading to a transient decrease in the airway pressure (figure 2*g*,*i*).

Eventually, the tips of the air finger reach the terminal acini, recruiting a first passage through the network (time marked with a cross in figure 2*g*–*i*), while recruited passages are illustrated with a filled circle at the terminus in figure 2*e*,*f*. Gradually, other passages are recruited and the airway pressure begins to rise as the prescribed volume increase now exceeds the local gain due to air finger elongation in individual airways. Once all the airways are recruited (figure 2*j*, filled circle in figure 2*g*–*i*), the continuous increase in air finger volume then drives a further rise in airway pressure. Eventually, the airway pressure exceeds the AOP (open circle on figure 2*g*), allowing the acini to open and triggering a significant increase in the total lung volume (figure 2*g*). Very similar dynamics were observed in 100 repeated runs of the model; the measured standard deviation over these 100 runs is shaded for the airway pressure (figure 2*i*) and the total number of airways recruited (figure 2*j*). In summary, this figure illustrates the temporal and spatial distribution of the air finger during ventilation of a collapsed airway network and demonstrates how an avalanche of airway recruitment manifests as a transient decrease in the airway pressure which can be identified on the pressure–volume trace, and identified with a release of elastic energy from the highly collapsed airway walls.

**Figure 3.**
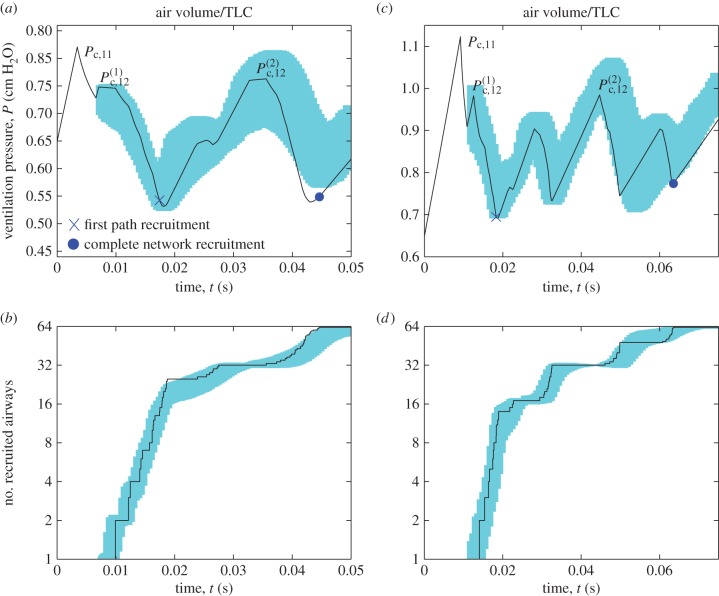
Summary of typical recruitment events for modest variability in airway cross-sectional area *d* = 0.2 and fixed tidal volume *V*_T_ = 10 ml kg^−1^. Modest collapse: (*a*) time-trace of airway pressure during airway recruitment for *C* = 0.5; (*b*) time-trace of the number of recruited airways. No collapse: (*c*) time-trace of airway pressure during airway recruitment for *C* = 1.0; (*d*) time-trace of the number of recruited airways for *C* = 1.0. Shaded areas in (*a–d*) correspond to 1 s.d. from the mean for 100 repeated simulations of the model. Other parameters are chosen as in table 1.

To assess how the dynamics of recruitment are modified when the network is less collapsed and hence more flooded, figure 3 illustrates typical time-traces of the airway pressure and number of recruited airways for the ventilation of networks where the airways are collapsed to 50% of their equilibrium cross-sectional area (*C* = 0.5, figure 3*a*,*b*) and where the airways are flooded but not collapsed (*C* = 1.0, figure 3*c*,*d*), each with modest variability in airway area across each generation (*d* = 0.2) and ventilated with the same protocol as in figure 2. Recall that since the air finger volume increases linearly in time in all cases (figure 2*h*), the corresponding pressure–volume curves will take an identical shape to figure 3*a*,*c*. In both cases, the initial airway pressure is below the critical opening pressure for the parent airway in generation 11 (denoted 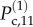), so the air finger cannot propagate and the upper airways must expand to accommodate the prescribed increase in volume, gradually raising the airway pressure (figure 3*a*,*c*). Eventually, the airway pressure is sufficiently large to initialize recruitment of generation 11, triggering a decrease in pressure as the air finger advances, falling below the threshold opening pressure for both daughters in generation 12. The air finger halts while the airway pressure increases again, until eventually the threshold opening pressure for one of the daughter airways in generation 12 is exceeded (denoted 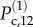). The subsequent avalanche of recruitment drives the airway pressure to decrease once again; this cycle of increase and decrease in the airway pressure continues until a first passage has been recruited to the acini (cross in figure 3*a*,*c*) and all the airways in the half of the network downstream of the open airway in generation 12 are recruited (figure 3*b*,*d*). The air finger must halt once again, and the airway pressure increases until eventually the opening pressure for the other airway in generation 12 is exceeded (denoted 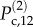), triggering a recruitment of the remainder of the network via two final avalanches (cross in figure 3*a*,*c*). Similar dynamics are exhibited in the 100 repeated simulations of the model, where 1 s.d. from the mean is shown as the shaded regions in figure 3*a*–*d*. In summary, this figure demonstrates how a reduction in the degree of airway collapse inhibits spontaneous recruitment, where instead the airways must be forced open via a number of distinct avalanches, increasing the total recruitment time significantly; the corresponding pressure–volume trace is also significantly more erratic than figure 2*d*. Early recruitment of the proximal airways in the network is therefore key to complete and rapid recruitment of the entire structure.

**Figure 4.**
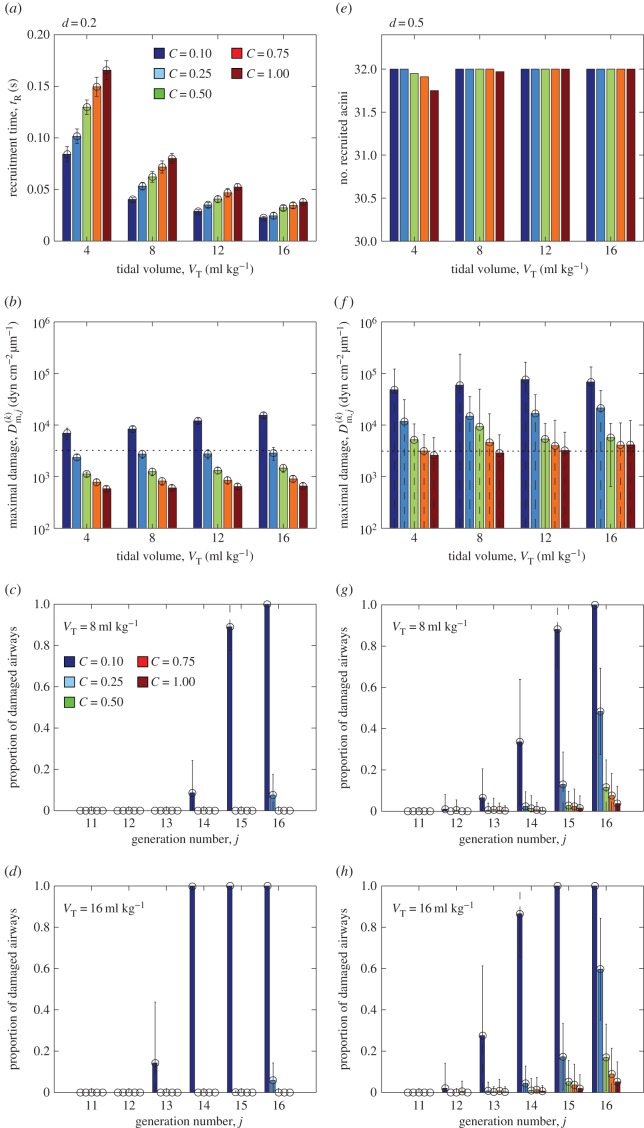
Summary of repeated model simulations for tidal volumes *V*_T_ = 4 ml kg^−1^ to *V*_T_ = 16 ml kg^−1^ and degree of collapse *C* = 0.1 to *C* = 1: (*a*) recruitment time for *d* = 0.2; (*b*) maximal pressure gradient in the liquid ahead of the air fingertip for *d* = 0.2; proportion of airways in each generation where the maximal pressure gradient in the liquid ahead of the tip exceeds the damage threshold 10^3.5^ dyn cm^−2^ µm^−1^ for *d* = 0.2 (note the logarithmic scale) with (*c*) *V*_T_ = 8 ml kg^−1^; (*d*) *V*_T_ = 16 ml kg^−1^; (*e*) mean number of acini recruited for *d* = 0.5; (*f*) maximal pressure gradient in the liquid ahead of the air fingertip for *d* = 0.5; proportion of damaged airways in each generation for *d* = 0.5 with (*g*) *V*_T_ = 8 ml kg^−1^; (*h*) *V*_T_ = 16 ml kg^−1^. Other parameters are chosen as in table 1.

To assess the influence of network heterogeneity on recruitment and ventilation, figure 4 illustrates the results from repeated simulations of the model for a range of tidal volumes *V*_T_ = 4 ml kg^−1^ to *V*_T_ = 16 ml kg^−1^ and degree of network collapse *C* = 0.1 to *C* = 1 for two different standard deviations in maximal cross-sectional area, *d* = 0.2 (figure 4*a–d*) and *d* = 0.5 (figure 4*e*,*f*), considering 100 runs in each case. These graphs further illustrate the standard deviation of the measured data using error bars. Note that in most simulations with large variability (*d* = 0.5), the distributions are strongly non-Gaussian and in some cases the standard deviation exceeds the mean; in these cases, the error bars lead to unphysical predictions and these are shown as a dashed line (e.g. a proportion of damaged airways in figure 4*g* that is greater than one or less than zero). For modest airway heterogeneity (*d* = 0.2), the mean time taken to recruit all the airways in the network (denoted 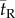) is shorter for larger tidal volumes (figure 4*a*). Recruitment is also faster for more collapsed networks, since the stored elastic energy in the collapsed wall helps to overcome the adhesive surface-tension forces (figure 4*a*). For simulations with *d* = 0.2, the entire network is always ultimately recruited over the ventilation cycle and the tidal volume is sufficiently low for the airway pressure to remain well below the peak pressure expected during normal respiration. However, the mean maximal pressure gradient in the liquid directly ahead of the air fingertip increases weakly for increasing tidal volumes and strongly for increasing degree of collapse (figure 4*b*), promoting damage to the cells lining the airway walls. To further examine the spatial distribution of injury, we characterize an individual airway as damaged when the mean maximal pressure gradient exceeds 10^3.5^ dyn cm^−2^ µm^−1^; this arbitrary threshold illuminates global trends. A histogram of the proportion of damaged airways in each generation is shown for different degrees of network collapse for *V*_T_ = 8 ml kg^−1^ (figure 4*c*) and *V*_T_ = 16 ml kg^−1^ (figure 4*d*). For low airway variability, the airway damage is restricted to the peripheral airways, with the proportion increasing for less collapsed networks (figure 4*c*) and for increasing tidal volumes (figure 4*d*).

For large airway heterogeneity (*d* = 0.5), the total time required to recruit the network is significantly increased. In all cases, considered the mean recruitment time across the 100 simulations is increased by a factor of at least 1.4, while in some cases the mean recruitment time can be as much as six times longer. The distribution of these recruitment times across the 100 simulations also becomes significantly non-Gaussian with large standard deviation, and in some cases the recruitment time is longer than the time interval over which ventilation is applied (*t*_R_ > *T*_Q_), so not all acini in the network (total 32) are ventilated and recruitment is incomplete (figure 4*e*). Increased heterogeneity in maximal cross-sectional area also results in an increased mean maximal pressure gradient in the liquid close to the tip of the air finger (figure 4*f*), increased by an approximate factor of 10 from that observed for *d* = 0.2 (figure 4*b*). This in turn leads to an increased proportion of damaged airways per generation (figure 4*g*,*h*). Larger variability in the distribution of airway stiffness leads to much larger standard deviations in the predictions of the maximal pressure gradient and hence airway damage. In all cases, networks that are flooded but not collapsed (*C* = 1) experience a significantly reduced maximal pressure gradient in the liquid ahead of the tip of the air finger compared to collapsed networks, leading to a significant reduction in airway damage. In summary, the model predicts that increased network heterogeneity increases the time taken for airway recruitment, decreases the likelihood of complete ventilation and results in a significantly increased pressure gradient exerted on the airway wall and hence an increased (and more widely dispersed) amount of airway damage.

**Figure 5.**
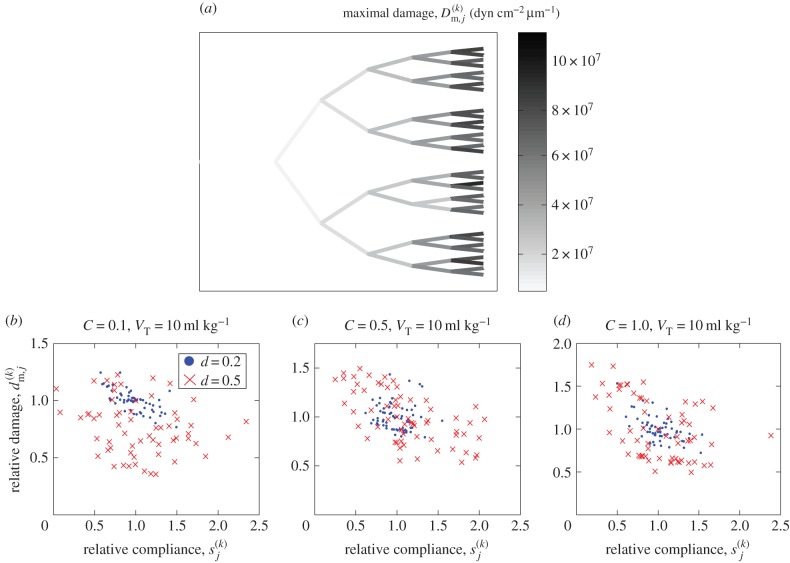
Relationship between the distribution of airway stiffness and the subsequent airway damage for the baseline simulation shown in figure 2: (*a*) the six-generation airway tree, shaded according to the maximal pressure gradient in the liquid during recruitment of that generation, providing a measure of airway damage. Scatterplots of the airway stiffness (*x*-axis) compared to the relative damage to that airway compared to the mean for that generation (*y*-axis) for particular examples with *V*_T_ = 10 ml kg^−1^ and *d* = 0.2 (filled circles) and *d* = 0.5 (crosses) for (*b*) *C* = 0.1; (*c*) *C* = 0.5; (*d*) *C* = 1.0.

To assess the spatial extent of the damage exerted on the airway wall during a typical recruitment event, figure 5 illustrates the maximal pressure gradient exerted on each airway (figure 5*a*) as a measure of airway damage for the baseline simulation shown in figure 2 (see also the electronic supplementary material, movie). This figure demonstrates that the maximal pressure gradient in the liquid ahead of the tip of the air finger is always greatest during recruitment of the peripheral airways (generation 16), suggesting that these airways sustain the most damage. Furthermore, to quantify the relationship between stiffness and damage, figure 5*b*–*d* shows scatterplots comparing the individual airway stiffness, 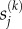, to the relative damage to that particular airway, 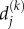, for all 63 airways in the network during individual recruitment events. These figures compare three different degrees of collapse (*C* = 0.1, figure 5*b*, *C* = 0.5, figure 5*c*, *C* = 1.0, figure 5*d*) for both low variability (*d* = 0.2, filled circles) and large variability (*d* = 0.5, crosses). These figures demonstrate no significant evidence of correlation between the airway stiffness and the relative damage, with Pearson correlation coefficients computed in the range −0.067 to −0.706 for these six examples. The outliers with large relative damage in figure 5*e* correspond to recruitment during a particular avalanche towards the end of the event with a large maximal pressure gradient in the liquid ahead of the tip of the air finger. In summary, this figure illustrates that airway damage is most significant in the peripheral airways of the network, but there is no evidence of correlation between the airway stiffness and damage during recruitment.

### 3.1. Influence of positive-end-expiratory pressure

**Figure 6.**
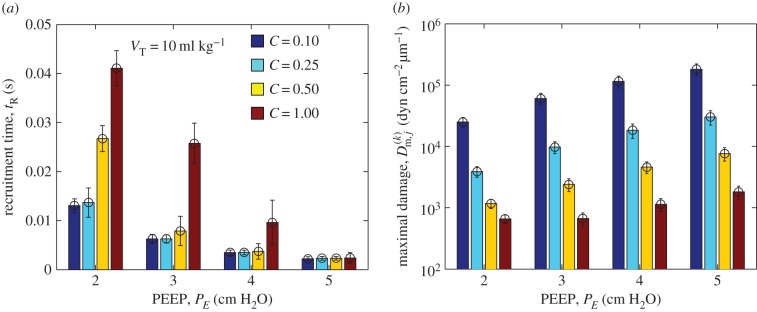
Repeated simulations of the model for low variability (*d* = 0.2) comparing recruitment with *V*_T_ = 10 ml kg^−1^ for *C* = 0.1 to *C* = 1 for various PEEP *P_E_* = 2 cm H_2_O to *P_E_* = 5 cm H_2_O: (*a*) mean recruitment time; (*b*) the mean maximal pressure gradient in the liquid ahead of the tip of the air finger (note the logarithmic scale). Other parameters are chosen as in table 1.

To assess the influence of a PEEP on recruitment, figure 6 illustrates a summary of 100 repeated simulations of the model for fixed tidal volume *V*_T_ = 10 ml kg^−1^ using a PEEP ranging from 2 cm H_2_O to 5 cm H_2_O for various degrees of airway collapse (*C* = 0.1 to *C* = 1) with modest variability in airway cross-sectional area (*d* = 0.2). The bar graphs illustrate the mean over these simulations, while the error bars represent the corresponding standard deviation. As the PEEP increases, the mean time taken to recruit all the airways in the network decreases significantly (figure 6*a*), particularly for airways that are less collapsed initially, while the mean maximal pressure gradient in the liquid ahead of the tip of the air finger increases, increasing the likelihood of damage to the constituent airways (figure 6*b*), the increase being greater for airways that are more collapsed initially. Thus, according to the model, PEEP decreases the recruitment time at the cost of greater damage to the cells lining the airway wall; however, the cost of recruitment (larger damage) is smallest for airways that are initially less collapsed (figure 6*b*), while the corresponding recruitment time of these less collapsed networks remains well below the time over which ventilation is applied.

## 4. Discussion

Recruitment of a flooded network of flexible airways has been studied using a theoretical model, incorporating a detailed understanding of the fluid mechanics associated with recruiting a single airway [14]. The model identifies a yield pressure dependent on the local elastic properties which must be exceeded before recruitment of an individual airway can take place and also accounts for a finite recruitment time for the tip of the air finger to propagate along the airway. The model incorporates anatomical measurements of the airway tree for adult humans [35], accounts for the change in airway compliance between generations [34] and also incorporates stochastic heterogeneity by sampling the mean cross-sectional area of individual airways from a Gaussian distribution centred about the mean for that generation, leading to a distribution of airway stiffness. The model neglects other sources of inhomogeneity in a human lung, such as variation in the length of airways across a generation or variable branching angles. The airway liquid lining is assumed to have a Newtonian rheology (non-Newtonian effects have been considered elsewhere [41]) and the coefficient of surface tension along the air–fluid interface is assumed constant (neglecting the influence of surfactant transport [42]). Gravitational effects are not considered in this study. Mechanical interdependence between adjacent airways is not accounted for, nor are the complicated patterns of air flow that could arise at an airway bifurcation [43]. Clearly, there is considerable scope for extending the model in future to account for these additional effects.

It should be noted that this study imposes a particular (and rather strong) covariance structure on the random variables in the model. Properties of individual airways (such as maximal extension, wall stiffness and equilibrium area) are strongly correlated to each other through the assumed ‘tube law’, whereas the airway properties across a generation vary independently. Additional sources of variation will need to be considered in future versions of the model.

For highly collapsed networks with low variability in airway stiffness, the model predicts that ventilation with fixed tidal volume leads to homogeneous opening of the network via a single avalanche of successive recruitment events, facilitated by stored elastic energy in the airway walls, accompanied by a transient decrease in the airway pressure (figure 2*g*). These predictions are qualitatively similar to the avalanches of recruitment predicted by simple network models of ventilation using a prescribed airway pressure, where threshold opening pressures of lung units are allocated at random [25,27] (indeed, the present model can also be used to simulate ventilation with fixed airway pressure, with very similar results). This recruitment event is representative of the first breath of a healthy infant, where shortly before birth the airways are collapsed by active pumping of the liquid through the airway walls. However, for less collapsed networks, the recruitment becomes increasingly heterogeneous and can eventually become incomplete if the recruitment time exceeds the interval over which ventilation is applied. Recruitment now occurs via several temporally separated avalanches (figure 3), for which the airway pressure transiently falls below the yield pressure required for recruitment of some airways in the network.

This theoretical model may provide a predictive tool for testing recruitment strategies (such as those tested clinically by [44]), elucidating, in particular, the role of tidal volume in dictating efficient recruitment. The model predicts that increasing tidal volume decreases the time taken to recruit the network, which can then lead to more complete recruitment in networks with large variability in the elastic properties. However, increasing tidal volume also weakly increases the maximal stress exerted on the epithelial cells lining the airway wall, particularly in the lung periphery (figures 4 and 5). These observations are consistent with reports of the benefits of lower tidal volumes by the ARDS network [10] and in a recent clinical study where a small increase in the applied tidal volume early in ARDS is associated with significantly increased mortality [45].

Furthermore, the theoretical model elucidates the role of PEEP, reducing the time to complete ventilation of the network, but at the expense of greater damage exerted on the epithelial cells lining the airway wall (figure 6); this damage is more severe for airways that are initially collapsed.

In all simulations of the model presented here, the degree of collapse is held constant across all airways in the network, but the approach could be extended to sample the initial pattern of airway collapse from CT images [46,47] as a method for selecting patient-specific recruitment strategies and estimation of PEEP [48]. However, this is deferred to future work.

## Supplementary Material

Supplementary information

## Data accessibility

The data for the model simulations presented in this paper can be accessed online (http://dx.doi.org/10.5525/gla.researchdata.187).
