# Supplementary material for: Patterns of recruitment and injury in a heterogeneous airway network model
Source: J R Soc Interface. 2015 Oct 6;12(111):20150523. doi: 10.1098/rsif.2015.0523 (PMC4614491; doi:10.1098/rsif.2015.0523)
Supplement: Supplementary information [file rsif20150523supp1.pdf]

Patterns of recruitment and injury in a heterogeneous airway network model: Supplementary material

Peter S. Stewart & Oliver E. Jensen

August 19, 2015

1 Summary of the model

We develop a lumped-parameter model for recruitment a network of flooded and possibly collapsed flexible airways. The model is developed based on a number of assumptions, which we briefly summarise here.

1. The airway network has dichotomous bifurcations over six generations mimicking generations 11-16 of the human lung.

2. Each airway can be modelled as a planar symmetric flexible-walled channel in cross-section, characterised by a transverse 'height' and effective 'width' in the planes orthogonal to the axis of the channel (mimicking a buckled configuration).

3. Airway dimensions across generations are taken from the anatomical measurements of Weibel (1963). In particular, we employ his estimates of the mean diameter of airways in a given generation and the total cross-sectional area of each generation at 75% total lung capacity. The latter is used to estimate the 'maximal' cross-sectional area of airways at a given generation.

4. The 'maximal' cross-sectional area of individual airways in each generation is prescribed. Natural variability within airways in a given generation is introduced by sampling their cross-sectional (height/width) aspect ratios from independent normal distributions.

5. The airway cross-sectional area is related to the transmural (internal-external) pressure through the nonlinear tube law proposed by Lambert *et al.* (1982), rationally capturing the change in airway compliance between generations. In addition, the pressure-area relation is modified to incorporate longitudinal tension.

6. Each airway in the network is initially collapsed to 100 C% of its equilibrium cross-sectional area and completely flooded with fluid, where C falls in the range $0.1 \leq C \leq 1$. For $C = 1$ the airways are flooded but not collapsed.

7. The network is mechanically ventilated by injecting a finger of air with a prescribed flow rate. The time-dependent airway pressure is continuous across each airway bifurcation.

8. The influence of surfactant transport on the gas-liquid interface is ignored and so the surface tension coefficient is constant.

9. Of the two steady modes of recruitment elucidated for a single airway, known as 'pushing' (where the air finger pushes a long column of fluid ahead of itself like a leaky piston) and 'peeling' (where the airway walls peel apart rapidly) (Halpern *et al.*, 2005), only 'peeling' motion can lead to appreciable airway recruitment. The recruitment speed can be calculated as a function of the airway pressure, derived in a manner identical to Jensen *et al.* (2002).

10. Recruitment of a particular airway takes place while the airway pressure exceeds a yield pressure, which is a function of the airway elastic properties. Once the airway pressure has fallen below this threshold pressure, recruitment in that airway halts until the airway pressure increases beyond the threshold again.

11. The generation of airways at the distal end of the network is assumed to open into a compliant compartment representing the airways in the respiratory zone and the alveoli. The relationship between the airway pressure and volume of this compartment is based on that derived by Salazar & Knowles (1964).

We now explain how these assumptions are expressed mathematically.

2 Model for recruitment of a bifurcating airway network

We consider generations 11-16 of a human lung using 'Model A' from Weibel (1963) which prescribes regular dichotomous branching of generations (Assumption 1).

Fully three-dimensional simulations of individual airway recruitment have shown that the surface-tension-driven collapse of a fluid-lined tube is strongly asymmetric (Heil, 1999*a*,*b*; Hazel & Heil, 2003), where the airway typically adopts a ribbon-like configuration downstream of the tip of the air finger (see Fig. S1a). To mimic a buckled configuration, in this model each airway is modelled as a planar symmetric flexible-walled channel in cross-section (see Fig. S1b), characterised by a transverse 'height' and effective 'width' in the planes orthogonal to the axis of the channel (Assumption 2). Airway k in generation j is denoted by subscript j and superscript (k). The notation for the present model is summarised in Table. S1.

Weibel (1963) (see also Weibel, 1991) measured the lengths and diameters of individual airways and total cross-sectional area of each generation using casts of human lungs taken at 75% total lung capacity (TLC). The mean length and diameter of airways in generations 11-16 and their total cross-sectional area at 75% TLC are listed in Table S2. Following Lambert *et al.* (1982), we consider this total cross-sectional area as the 'maximal' airway area for all the airways at a given generation, denoted as $A_{m,j}$.

2.1 Estimation of the maximal airway cross sectional area

The mean maximal cross-sectional area for an individual airway in generation j, denoted $\overline{a}_{m,j}$, is assumed to take the form

$$\overline{a}_{m,j} = A_{m,j} 2^{-j}. \tag{1}$$

Hence, we can write the total cross-sectional area of generation j at any time t as

$$A_j = \sum_{k=1}^{2^j} a_j^{(k)}. \tag{2}$$

To account for natural variability between airways across a generation we sample the maximal cross-sectional area for each individual airway $(a_{m,j}^{(k)})$ relative to the mean for that generation

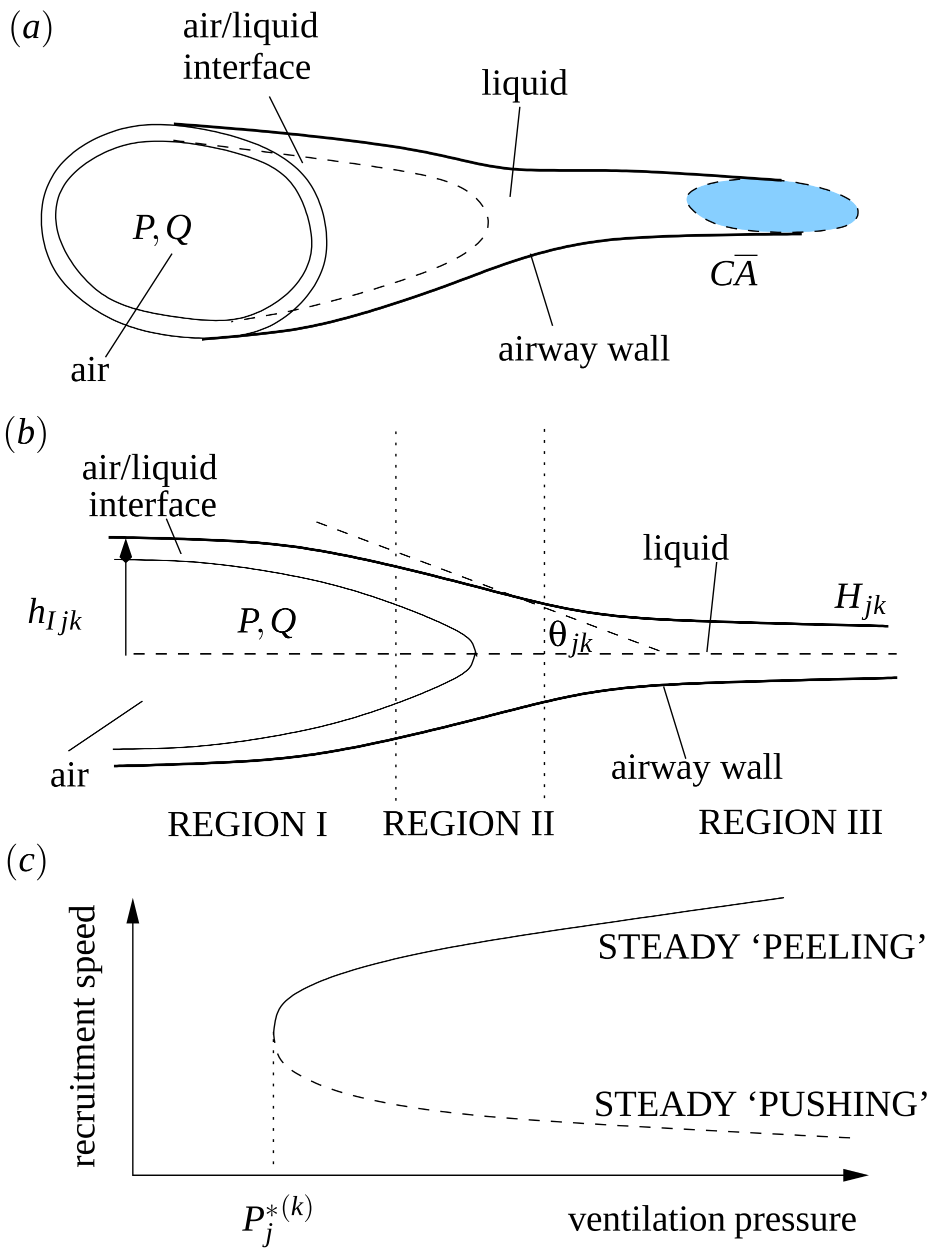

Figure S1: Schematic of recruitment of a single airway: (a) three-dimensional sketch of airway recruitment showing the buckled 'ribbon-like' configuration Hazel & Heil (2003); the cross-section shape is characterised by and effective 'width' z and a transverse 'height' $2h$. (b) cross section of a single airway, resembling 2D flexible walled channel models Halpern *et al.* (2005); (c) the relationship between airway pressure and recruitment speed for steady recruitment of a single airway.

($\overline{a}_{m,j}$). In particular we define the relative stiffness parameter $s_j^{(k)} = a_{m,j}^{(k)}/\overline{a}_{m,j}$ and sample its value from a normal distribution with unit mean and variability d (Assumption 4)

$$s_j^{(k)} \sim \mathrm{N}(1, d^2). \tag{3}$$

Quantity	
Symbol	Global properties
$P(t)$	airway pressure
p	airway liquid pressure
Q	prescribed flow rate of air finger
γ	uniform surface tension
μ	uniform liquid viscosity
P_0	threshold opening pressure of alveoli (TOP)
T_0	constant longitudinal tension applied across the network
	Properties of generation j
A_j	total cross-sectional area of airways
$A_{m,j}$	maximal total cross-sectional area of airways
α_j	ratio of A_j to $A_{m,j}$
$a_{m,j}$	maximal cross-sectional area of individual airways
$h_{m,j}$	mean maximal transverse displacement of cross-section for airways
α_{0j}	ratio of equilibrium cross-sectional area to maximal
α'_{0j}	gradient of pressure-volume curve at zero transmural pressure (equilibrium)
T_j	longitudinal tension of airways
	Properties of airway k in generation j
$\tilde{U}_j^{(k)}$	pushing speed of recruitment
$a_j^{(k)}$	cross-sectional area
$h_j^{(k)}$	transverse dimension of cross-section
$h_{m,j}^{(k)}$	maximal traverse dimension of cross-section
$a_{m,j}^{(k)}$	maximal cross-sectional of an individual airway
$s_j^{(k)}$	ratio of maximal cross-sectional area to the mean for that generation
$h_{e,j}^{(k)}$	equilibrium traverse dimension of cross-section
$z_j^{(k)}$	effective width
$L_j^{(k)}(t)$	length of air finger
$U_j^{(k)}(t)$	peeling speed of recruitment
$h_{I,j}^{(k)}(t)$	transverse dimension of cross-section in Region I
$L_{I,j}^{(k)}$	length of asymptotic Region I
$L_{III,j}^{(k)}$	length of asymptotic Region III
$H_j^{(k)}$	downstream (collapsed) traverse dimension of airway cross-section
$\theta_j^{(k)}$	peeling angle

Table S1: Notational choices for the airway network model.

An estimate of the transverse dimensions of the airway can then be calculated assuming an approximately circular cross-section of radius $h_{m,j}^{(k)} = (a_{m,j}^{(k)}/\pi)^{1/2}$. In addition, we prescribe the effective 'width' of each airway across the tree as

$$z_j^{(k)} = a_{m,j}^{(k)}/(2h_{m,j}^{(k)}). \tag{4}$$

j	$2h_{m,j}$ (cm)	L_j(cm)	$A_{m,j}$ (cm^2)	α_{0j}	α'_{0j}	n_{1j}	n_{2j}
11	0.109	0.39	19.6	0.08	0.202	1.0	9.0
12	0.094	0.33	28.8	0.063	0.214	1.0	8.0
13	0.082	0.27	44.5	0.049	0.221	1.0	8.0
14	0.074	0.23	69.5	0.039	0.228	1.0	8.0
15	0.066	0.20	113.0	0.031	0.234	1.0	7.0
16	0.060	0.17	180.0	0.024	0.238	1.0	7.0

Table S2: Parameter choices generations 11-16 of a human lung, calculated based on Weibel (1963) and Lambert *et al.* (1982); Lambert & Beck (2004).

2.2 Model for airway wall elasticity

Lambert *et al.* (1982) proposed a relationship between transmural pressure across the airway wall and total airway cross-sectional area (like a 'tube law') for generations 0-16 of a human lung (see also Lambert & Beck, 2004). They modelled the expansion and collapse of each generation of airways based on anatomical data (Weibel, 1963, see Table S2). Each generation is characterised by an equilibrium area (at zero transmural pressure) and a maximal area (arising at infinite transmural pressure), recognising that airways stiffen when strongly inflated. The total collective equilibrium (or undisturbed) cross-sectional area of a particular generation is denoted as $\alpha_{0,j}A_{m,j}$ (where $\alpha_{0,j}$ is also listed in Table S2). In particular, this model describes the relationship between α_j, the total cross-sectional area of all the airways at generation j normalised on the the maximal cross-sectional area for that generation ($\alpha_j = A_j/A_{m,j}$), and the transmural pressure P. They used two hyperbolae to describe the inflation and collapse of each generation, appropriately matched together at $P = 0$. For all the airways at generation j

$$\alpha_j = F_j(P) = \begin{cases} \alpha_{0j}\left(1 - \frac{P}{P_{1j}}\right)^{-n_{1j}}, & P \leq 0, \\ 1 - (1-\alpha_{0j})\left(1 - \frac{P}{P_{2j}}\right)^{-n_{2j}}, & P \geq 0, \end{cases} \tag{5a}$$

where n_{1j} and n_{2j} are constants (tabulated in Table S2) and

$$P_{1j} = \frac{\alpha_{0j}n_{1j}}{\alpha'_{0j}}, \qquad P_{2j} = \frac{-n_{2j}(1-\alpha_{0j})}{\alpha'_{0j}}, \tag{5b}$$

where α'_{0j} represents $\mathrm{d}\alpha_j/\mathrm{d}P$ as $P \to 0^{\pm}$.

The relationship between α_j and P is plotted in Fig. S2(a) for generations 11-16. Similarly, the pressure-area curve for an individual airway in generation 14 with maximal area $\overline{a}_{m,j}$ is shown as a solid line in Fig. S2(b). Also shown are the pressure-area curves for airways with maximal area $(1 \pm 0.1)\overline{a}_{m,j}$. The corresponding compliance of this airway is the gradient of the P against a_j curve evaluated at $P = 0$. This figure illustrates that an increase in the maximal area of an airway corresponds to a decrease in compliance.

Using this notation, the equilibrium transverse 'height' of the cross-section in an individual airway can be expressed

$$h_{e,j}^{(k)} = \alpha_{0j}h_{m,j}^{(k)}, \tag{6}$$

ensuring (from (4)) that $z_j^{(k)}h_{e,j}^{(k)} = \alpha_{0j}a_{m,j}^{(k)}$ (no sum over j).

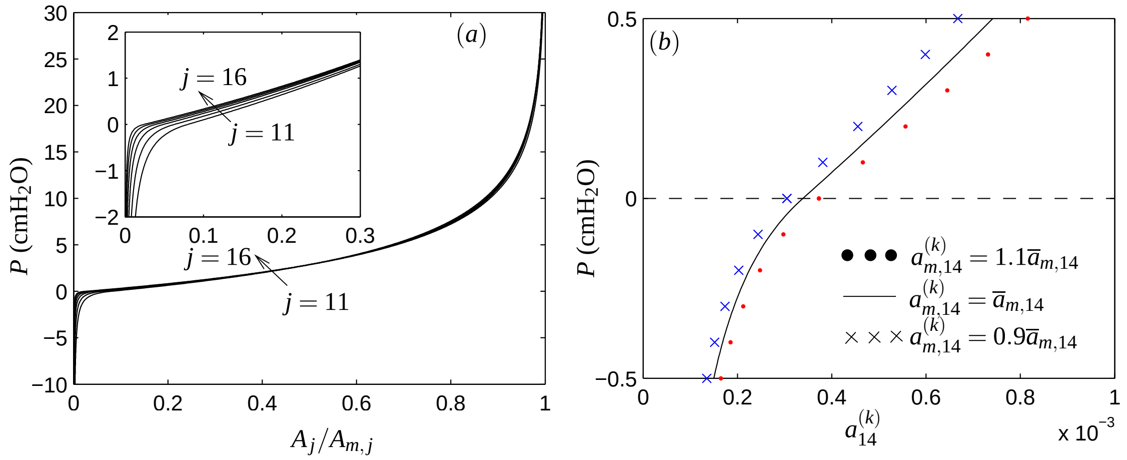

Figure S2: The variation in α_j, the total generation cross-sectional area A_j normalised on the 'maximal' cross-sectional area $A_{m,j}$, with P, the airway pressure, for generations 11-16. Based on data taken from Lambert *et al.* (1982), listed in Table S2.

Representing each airway as a two-dimensional channel, Lambert's tube law for generation j (5) can be re-expressed in the form

$$h_j^{(k)} = h_{m,j}^{(k)} F_j(P) = \left(\frac{\overline{a}_{m,j}^{(k)} s_j^{(k)}}{\pi} \right)^{1/2} F_j(P), \tag{7}$$

where $h_j^{(k)}$ is the transverse displacement of the cross-section for airway k in generation j (Assumption 5).

In addition, a constant longitudinal wall tension T_0 is applied between the upstream and downstream ends of the network. This tension is assumed to be constant for all the airways across a given generation, denoted T_j, and is calculated using a simple force balance around each bifurcation. If η is the symmetric branching angle, this implies $T_j = 2T_{j+1}\cos(\eta)$. Assuming small branching angles ($\eta \ll 1$) and neglecting external tethering forces, $T_j \approx 2T_{j+1}$.

2.3 Model for airway collapse

We assume that each airway cross-section (Fig. S1) constitutes two (finite-length) flexible sheets confining a region of Newtonian liquid with viscosity μ (Fig. S1). Initially each constituent airway is collapsed and each sheet is uniform, held a constant distance of $2H_j^{(k)}$ apart by the liquid (Assumption 6). For simplicity we place an axis midway between (and parallel to) the undeformed sheets, about which we assume the motion is symmetric. Throughout this work we assume that the $H_j^{(k)}$ is $100C\%$ of the equilibrium transverse 'height' of the airway wall ($h_{e,j}^{(k)}$), where C is a constant. Thus, $0 < C \leq 1$ measures the degree of initial airway collapse. In the main paper we investigate the interval $0.1 \leq C \leq 1$. The coordinate x measures distance along the airway.

A finger of air is introduced at the upstream end of the network with a prescribed flow rate Q (Assumption 7); the corresponding airway pressure is denoted $p_b(t)$ where t is time. The finger of air inflates the airway walls, forming an interface with the liquid with constant surface

tension γ. This surface tension coefficient is assumed constant, so the effects of surfactant transport through the network are ignored (Assumption 8). Neglecting the pressure drop due to the surface tension of the liquid (assumed to be much less than the wall tension, Halpern *et al.* (2005)) implies that $p_b = P$. Henceforth we denote the airway pressure as $P(t)$.

2.4 Steady recruitment

When the airway pressure exceeds a threshold for an individual airway, denoted $P_j^{*(k)}$, steady recruitment proceeds through one of two modes, known as 'pushing' (where the air finger pushes a long column of fluid ahead of itself like a leaky piston) and 'peeling' (where the airway walls peel apart rapidly) (Halpern *et al.*, 2005), as illustrated schematically in Fig. S1(c). Previous work considering the recruitment of a bifurcated airway has shown that for sufficiently high flow rates the system can sustain fast quasi-steady peeling motion in both daughter airways. For lower flow rates (where simultaneous steady peeling of both daughter airways cannot be sustained) the finger tip in one or both of the daughter airways makes the transition to slow pushing motion once the pressure falls below the critical pressure required to sustain steady motion. For simplicity, since peeling speeds greatly exceed pushing speeds, we assume that the finger of air exhibits quasi-steady peeling in any daughter airway where the pressure exceeds this critical threshold pressure (Assumption 9). Once the airway pressure falls below this threshold in a particular airway, we assume that recruitment of that airway stops until the airway pressure rises above this threshold again (Assumption 10).

We follow Jensen *et al.* (2002) in constructing a scaling approximation for the recruitment speed in peeling motion which can be implemented for each airway in the network (Assumption 9), dividing the flow into three asymptotic regions (Fig. S1b).

In region I we assume that $P \geq 0$ and the airway wall is inflated to a transverse 'height' $h_{I,j}^{(k)}$ (dependent on P) and in local equilibrium (we assume $h_{e,j}^{(k)} < h_{I,j}^{(k)} < h_{m,j}^{(k)}$). Modifying (7b), we express the normal stress balance across the airway wall as

$$P = P_{2j}\left(1 - \left(\frac{(1 - h_j^{(k)}/h_{m,j})}{(1-\alpha_{0j})}\right)^{-1/n_{2j}}\right) - T_j \frac{\partial^2 h_j^{(k)}}{\partial x^2}, \quad (P \geq 0), \tag{8}$$

combining Lambert's tube law with a linearised expression for the effect of airway wall tension. The transverse 'height' of the airway wall far upstream of the tip of the air finger, where $h_j^{(k)}$ is independent of x, is given by

$$h_{I,j}^{(k)} = h_{m,j}\left(1 - (1-\alpha_{0j})\left(1 - \frac{P}{P_{2j}}\right)^{-n_{2j}}\right), \quad (P \geq 0). \tag{9}$$

Across region I we balance the elastic restoring force with the airway wall tension, represented by the two terms on the RHS of (9). This balance implies that the scale of variation within region I can be expressed approximately as

$$L_{I,j}^{(k)} = \left[\frac{T_j h_{I,j}^{(k)}}{P_{2j}\left(1 - \left((1 - h_{I,j}^{(k)}/h_{m,j})/(1-\alpha_{0j})\right)^{-1/n_{2j}}\right)}\right]^{1/2}, \tag{10}$$

from which we can estimate the peeling angle (Fig. S1b)

$$\theta_j^{(k)} = h_{I,j}^{(k)}/L_{I,j}^{(k)}. \tag{11}$$

Region II contains the tip of the air finger and the flow field is complicated, but Halpern *et al.* (2005) have shown that it can be assumed passive provided the flux of fluid passing the tip and the corresponding pressure drop are accounted for.

Across region III (of approximate length $L_{III,j}^{(k)}$) the airway wall collapses to a transverse 'height' $H_j^{(k)}$ downstream. We choose this to be a fixed fraction, C, of the equilibrium transverse displacement of the airway, so $H_j^{(k)} = Ch_{e,j}^{(k)}$. Throughout this work we assume that C is a constant across the network (Assumption 6). Following Jensen *et al.* (2002), we estimate the pressure gradient in the long expanded region ahead of the finger tip from lubrication theory as

$$\frac{p}{L_{III,j}^{(k)}} \sim \frac{\mu U_j^{(k)}}{{H_j^{(k)}}^2}, \tag{12}$$

where p is the liquid pressure in region III.

The airway wall tension is assumed large enough to dominate the negative transmural pressure induced by bending of the airway wall, implying

$$p \sim \frac{T_j H_j^{(k)}}{{L_{III,j}^{(k)}}^2}, \tag{13}$$

so a second estimate of the peeling angle $\theta_j^{(k)}$ takes the form

$$\theta_j^{(k)} \sim H_j^{(k)}/L_{III,j}^{(k)} \sim \left(\mu U_j^{(k)}/T_j\right)^{1/3}. \tag{14}$$

Equating the two estimates (11,14) of the peeling angle $\theta_j^{(k)}$ gives an expression describing steady 'peeling' speed for airway k in generation j, relating the airway pressure P to the speed of the recruitment $U_j^{(k)}$:

$$U_j^{(k)} = \frac{1}{\mu}\left(\frac{h_{I,j}^{(k)} P_{2j}\left(1-\left((1-h_{I,j}^{(k)}/h_{m,j})/(1-\alpha_{0j})\right)^{-1/n_{2j}}\right)}{T_j^{1/3}}\right)^{3/2}, \tag{15}$$

where $h_{I,j}^{(k)}$ depends non-linearly on P. However, P must exceed a threshold in each daughter airway, which we denote $P_{jk}^{\star}$.

2.5 The yield pressure for airway recruitment

A suitable description of the speed of airway recruitment in steady pushing motion (denoted $\tilde{U}_j^{(k)}$) is required to derive an estimate of the critical pressure for steady recruitment in an individual airway $P_j^{*(k)}$ (where the steady pushing and peeling branches intersect, discussed Halpern *et al.* (2005) and illustrated schematically in Fig. S1(c)). The upstream transverse displacement of the airway wall can again be approximated using (9), so the total flux of liquid lost across the meniscus (according to Bretherton, 1961) takes the form

$$q_{u,jk} = \alpha_s \left(\frac{\mu \tilde{U}_j^{(k)}}{\gamma}\right)^{2/3} h_{I,j}^{(k)} \tilde{U}_j^{(k)}, \tag{16}$$

where $\tilde{U}_j^{(k)}$ is the pushing speed and $\alpha_s \approx 1.337$. The total volume of fluid swept up ahead of the advancing air finger tip takes the form

$$q_{d,jk} = \tilde{U}_j^{(k)} C h_{e,j}^{(k)}. \tag{17}$$

For steady pushing these two fluxes balance exactly and thus we derive that

$$\tilde{U}_j^{(k)} = \frac{\gamma}{\mu} \left(\frac{C h_{e,j}^{(k)}}{\alpha_s h_{I,j}^{(k)}} \right)^{3/2}. \tag{18}$$

The critical pressure required for recruitment a particular airway occurs when steady pushing and peeling intersect, so $P_{jk}^{\star}$ is a solution of

$$\gamma^{2/3} \left(\frac{C h_{e,j}^{(k)}}{\alpha_s h_{I,j}^{(k)}} \right) = \left(\frac{h_{I,j}^{(k)} P_{2j} \left(1 - \left((1 - h_{I,j}^{(k)}/h_{m,j})/(1-\alpha_{0j}) \right)^{-1/n_{2j}} \right)}{T_j^{1/3}} \right), \tag{19}$$

calculated numerically using Newton's method implemented in MATLAB. For the simulations shown we only accepted solutions which MATLAB returns as converged within a tolerance of 10^{-8}. In these simulations we used an initial pressure of 0.5545 cm $H_2 0$ and an initial speed of 0 cm s^{-1} for each airway, but found that the solver was not sensitive to the choice of initial conditions. We performed a variety of numerical experiments starting with a wide range of randomised initial conditions; simulations which MATLAB reported as converged always attained the same value.

The critical recruitment pressure for each generation using our benchmark parameters (Table S3 below) is shown in Fig. 1(d) in the main paper.

2.6 Model for alveolar compliance

We assume that the airways in the most distal generation considered (generation 16 in this case) open into a compliant bag representing the airways in the respiratory zone and the alveoli (Assumption 11). Following Bates & Irvin (2002), we assume that this sac will expand and contract with changing P, according to the Salazar–Knowles relationship (Salazar & Knowles, 1964),

$$V(t) = V_A \left(1 - \mathrm{e}^{-K_A P(t)} \right), \tag{20}$$

where V_A is the maximal volume of each acinar compartment and K_A is a compliance parameter Bates & Irvin (2002). This model has zero acinar volume for $P = 0$.

We modify this model to allow the alveoli to accommodate a theoretical opening pressure (TOP) for the alveoli P_A such that

$$V(t) = \begin{cases} V_A \left(1 - \mathrm{e}^{-K_A(P-P_A)} \right), & (P > P_A), \\ 0, & (P < P_A). \end{cases} \tag{21}$$

2.7 Ventilating the airway network

As in previous compartmental models (Halpern *et al.*, 2005), we assume that in each airway being recruited the rate of lengthening of the air finger is equal to the recruitment speed identified in (15), so that

$$\frac{\mathrm{d}}{\mathrm{d}t} L_j^{(k)} = U_j^{(k)}. \tag{22}$$

Parameter	value	units	source
T	3000	$\mathrm{dyn\,cm^{-1}}$	Naire & Jensen (2005)
γ	30	$\mathrm{dyn\,cm^{-1}}$	Kamm (1999)
μ	0.01	$\mathrm{dyn\,s\,cm^{-2}}$	Naire & Jensen (2005)
K_A	0.14	$\mathrm{cmH_20^{-1}}$	Bates & Irvin (2002)
V_A	0.0778	$\mathrm{cm^3}$	Prediletto *et al.* (2007)
P_A	2	$\mathrm{cmH_2O}$	Hickling (2002)
P_E	$2-5$	$\mathrm{cmH_2O}$	

Table S3: Parameter choices for an adult human lung.

The air finger is inflated with a prescribed flow rate, so the corresponding airway pressure will change with time. The total air finger volume has three contributions: the volume of the upper airways that are always open (generations 0-10), then open (or partially open) airways in generations 11-16 and the open alveoli. The total flow rate Q is balanced by

$$Q = \frac{\mathrm{d}}{\mathrm{d}t}\left(\sum_{j=0}^{10} A_j L_j + \sum_{j\geq 11} 2h_{I,j}^{(k)} z_j^{(k)} L_j^{(k)} + \sum_l H_l(P-P_A)V_A\left(1-\mathrm{e}^{-K_A(P-P_A)}\right)\right), \quad (23)$$

where l is an index over all the acini and the function H_l is a Heaviside function, which is set to zero for $P < P_A$ and set to one for $P \geq P_A$. Eqn. (23) can be rearranged to form an ODE for $\mathrm{d}P/\mathrm{d}t$ in terms of P, $U_j^{(k)}$, $L_j^{(k)}$ and the model parameters.

2.8 Simulating the model numerically

For M airways being recruited at time t, we have a system of $2M$ equations ((15) and (22) for each airway for the M quasi-steady recruitment speeds and M lengths of bubble fingers). These are coupled to the pressure condition (23) forming a system of $2M+1$ equations. These governing equations were solved using MATLAB solver `ode15s` for differential-algebraic equation systems. A typical simulation for a 6 generation network takes approximately 2 minutes on a desktop computer. In simulations we employed an absolute tolerance of 10^{-6} and a relative tolerance of 10^{-4}. Selected simulations have been validated against equivalents using error tolerances a factor of 10 smaller (everything else held the same) and the time-traces of airway pressure were almost indistinguishable.

To complete the description of the numerical model we specify an initial condition for the airway pressure $P(t)$. In this study we explored two possible initial conditions for the system.

The first choice was to assume that theme that the bubble is peeling steadily in the parent generation, with the airway pressure and peeling speed determined directly from the choice of prescribed volume flux, calculated from (19) for $j = 11$ using Newton's method (with an absolute tolerance of 10^{-8} and a relative tolerance of 10^{-8}).

Alternatively, the second choice was to prescribe a positive end expiratory pressure (PEEP) during recruitment, which maintained that the airway pressure must always exceed a particular value P_E; in simulations of the model including a PEEP we prescribed the initial pressure as $P = P_E$.

References

Bates, JHT & Irvin, CG 2002 Time dependence of recruitment and derecruitment in the lung: a theoretical model. *J. Appl. Physiol.* **93** (2), 705.

Bretherton, FP 1961 The motion of long bubbles in tubes. *J. Fluid Mech.* **10**, 166–188.

Halpern, D, Naire, S, Jensen, OE & Gaver, DP 2005 Unsteady bubble propagation in a flexible channel: predictions of a viscous stick-slip instability. *J. Fluid Mech.* **528**, 53–86.

Hazel, AL & Heil, M 2003 Three-dimensional airway reopening: the steady propagation of a semi-infinite bubble into a buckled elastic tube. *J. Fluid Mech.* **478**, 47–70.

Heil, M 1999*a* Airway closure: Occluding liquid bridges in strongly buckled elastic tubes. *ASME J. Biomech. Engng.* **121**, 487–493.

Heil, M 1999*b* Minimal liquid bridges in non-axisymmetrically buckled elastic tubes. *J. Fluid Mech.* **380**, 309–337.

Hickling, KG 2002 Reinterpreting the pressure-volume curve in patients with acute respiratory distress syndrome. *Curr. Opin. Crit. Care* **8** (1), 32–38.

Jensen, OE, Horsburgh, MK, Halpern, D & Gaver III, DP 2002 The steady propagation of a bubble in a flexible-walled channel: Asymptotic and computational models. *Phys. Fluids* **14**, 443–457.

Kamm, RD 1999 Airway Wall Mechanics. *Ann. Rev. Biomed. Engng.* **1** (1), 47–72.

Lambert, RK & Beck, KC 2004 Airway area distribution from the forced expiration manoeuvre. *J. Appl. Physiol.* **97** (2), 570–578.

Lambert, RK, Wilson, TA, Hyatt, RE & Rodarte, JR 1982 A computational model for expiratory flow. *J. Appl. Physiol.* **52** (1), 44–56.

Naire, S & Jensen, OE 2005 Epithelial cell deformation during surfactant-mediated airway reopening: a theoretical model. *J. Appl. Physiol.* **99** (2), 458–471.

Prediletto, R, Fornai, E, Catapano, G & Carli, C 2007 Assessment of the alveolar volume when sampling exhaled gas at different expired volumes in the single breath diffusion test. *BMC Pulmonary Med.* **7** (1), 18.

Salazar, E & Knowles, JH 1964 An analysis of pressure-volume characteristics of the lungs. *J. Appl. Physiol.* **19** (1), 97.

Weibel, ER 1963 Morphometry of the human lung. In *Academic*. Springer, Berlin.

Weibel, ER 1991 Design of airways and blood vessels considered as branching trees. In *The Lung: Scientific foundations* (ed. RG Crystal & JB West), pp. 711–720. New York: Raven.
